# Epistaxis during the third trimester of pregnancy is associated with blood transfusion a retrospective case–control study

**DOI:** 10.1007/s00404-026-08334-1

**Published:** 2026-02-11

**Authors:** Aviad Sapir, Lior Friedrich, Yonathan Osovizky, Yotam Heilig, Oded Cohen, Shay Schneider

**Affiliations:** 1 Department of Otolaryngology-Head and Neck Surgery, Soroka University Medical Center, 84101 Beer-Sheva, Israel; 2 Faculty of Health Sciences, Ben-Gurion University, Beer-Sheva, Israel; 3 Rabin Medical Center, Sackler Faculty of Medicine, Helen Schneider Hospital for WomenBeilinson HospitalTel Aviv University, Tel Aviv, Israel; 4 Clinical Research Center, Soroka University Medical Center, Beer-Sheva, Israel; 5 Department of Otolaryngology-Head and Neck Surgery, Samson Assuta Ashdod University Hospital, Ashdod, Israel

**Keywords:** Epistaxis, Pregnancy, Third-trimester bleeding, Blood transfusion, Preterm labor

## Abstract

**Background:**

Epistaxis is common during pregnancy due to physiological changes, yet its clinical significance regarding obstetric outcomes is poorly understood. This study investigated the associations between epistaxis during pregnancy and maternal and neonatal outcomes.

**Methods:**

We conducted a retrospective case–control study (2013–2022) at a single tertiary medical center. The study group included 104 pregnant women presenting with epistaxis, matched with 1924 controls based on age, ethnicity, and preexisting comorbidities. Multivariable logistic regression was used to identify independent predictors of adverse outcomes, including blood transfusion and preterm labor.

**Results:**

Women with epistaxis experienced significantly higher rates of third-trimester vaginal bleeding (7.7% vs. 1.1%; p < 0.001), preterm labor (15.4% vs. 8.7%; p = 0.022), and blood transfusion requirements (4.8% vs. 1.6%; p = 0.014). In a multivariable model, third-trimester epistaxis emerged as an independent predictor for blood transfusion (OR 4.96, 95% CI 1.47- 14.38; p = 0.005), even after adjusting for delivery mode and initial hemoglobin levels. While univariate analysis associated epistaxis with preterm labor, this relationship did not remain significant in the multivariable model (p = 0.254). Most epistaxis episodes (81.7%) were mild and resolved spontaneously.

**Conclusion:**

Epistaxis during pregnancy, particularly in the third trimester, is independently associated with a nearly fivefold increase in the odds of requiring a blood transfusion. While typically considered benign, epistaxis may serve as a clinical marker for systemic vascular susceptibility. These findings suggest that pregnant women presenting with epistaxis may benefit from enhanced clinical surveillance and interdisciplinary coordination to manage potential peripartum hemorrhagic complications.

**Supplementary Information:**

The online version contains supplementary material available at 10.1007/s00404-026-08334-1.

## What does this study add to the clinical work?

**Table:** 

This study highlights third-trimester epistaxis as a potential warning sign for significant peripartum bleeding. Incorporating this symptom into risk assessment may improve preparedness for hemorrhagic complications at delivery.	

## Introduction

Epistaxis affects approximately 6% of individuals seeking medical attention in the United States [1]. It exhibits a bimodal age distribution, peaking in children under 10 years and adults aged 45- 65 years, with no significant sex differences [2]. In pregnant women, epistaxis is often attributed to the substantial physiological adaptations associated with gestation [3].

While epistaxis is well-characterized in the general population, its incidence, risk factors, and clinical consequences during pregnancy remain poorly understood. Current data are largely limited to case reports, though one survey-based study reported a higher prevalence of epistaxis among pregnant women and an associated risk of postpartum hemorrhage (PPH) [4]. Various physiological changes during pregnancy and the puerperium- including increased susceptibility to viral infections, pregnancy-induced rhinitis, hypercoagulability, and hypertensive disorders- may contribute to epistaxis [5, 6]. Despite these observations, evidence regarding subsequent peripartum complications remains scarce.

The present study is the first to systematically examine associations between epistaxis during pregnancy and both maternal and neonatal outcomes. We also investigate potential risk factors, such as third-trimester bleeding and blood transfusion requirements. By clarifying the clinical significance of epistaxis in pregnancy, this study aims to inform obstetric management and improve maternal care.

## Methods

### Study design and setting

We conducted a retrospective case–control study of women who presented to the emergency room (ER) with epistaxis and subsequently delivered within nine months, between 2013 and 2022, at Soroka University Medical Center (SUMC), Beer Sheva, Israel. The study was approved by the institutional ethics committee (0361-22-SOR).

### Exposure definition and study groups

The exposed group included women presenting to the ER with a primary complaint of epistaxis. Controls were retrospectively selected from the hospital delivery database and delivered during the same period (2013–2022). Controls were matched to cases at a ratio of approximately 1:20 based on age, ethnicity (Jewish or Bedouin), history of primary hypertension, rhinitis (allergic or chronic), and known coagulopathies. Women with a history of hematologic disorders, prior nasal or sinus surgery, or anticoagulation therapy during pregnancy were excluded. Cases with incomplete documentation regarding pregnancy or labor were also excluded.

### Data collection

Data collected included medical history (rhinitis [allergic or chronic], primary hypertension, and coagulopathy) and details of epistaxis management, including local pressure, nasal packing, diathermy, or surgical intervention. Hemoglobin (g/dL; normal range 12–16) and white blood cell count (WBC; 103/µL; normal range 4.8–10.8) were recorded at the index epistaxis event for cases and at delivery for all participants.

### Outcomes

Maternal and neonatal outcomes included gestational diabetes mellitus (DM), gestational hypertension, vaginal bleeding during the second or third trimester, pre-eclampsia and eclampsia, preterm labor, preterm rupture of membranes (PROM), polyhydramnios or oligohydramnios, mode of delivery (vaginal or cesarean section), length of hospitalization, Apgar scores at 1 and 5 min, PPH, and requirement for blood transfusion.

### Statistical analysis

Descriptive statistics were used to summarize all variables. Continuous variables are reported as medians with interquartile ranges (Q1–Q3) and means with standard deviations, as appropriate. Categorical variables are presented as counts and percentages. Between-group comparisons were performed using 95% confidence intervals and/or p-values. The Mann–Whitney test was used for non-normally distributed continuous variables, and chi-square or Fisher’s exact tests were applied for categorical variables, depending on cell size.

Variables for multivariable models were selected based on clinical relevance and statistical significance, including third-trimester bleeding, preterm labor, cervical incompetence, blood transfusion, parity, hospitalization length, cesarean section, and maternal age. Conditional logistic regression models were used to assess associations. To avoid multicollinearity, variables with Spearman correlation coefficients > 0.7 were excluded. All analyses were performed in RStudio (version 1.1.423), with p < 0.05 considered statistically significant.

## Results

The study included 2028 pregnant women: 104 in the epistaxis group and 1924 in the control group. Demographic and clinical characteristics are summarized in Table 1. Age and ethnicity were similar between groups. Parity was lower among women with epistaxis (median 1.0 [IQR 0- 2.0] vs. 1.0 [0- 3.0]; p = 0.016), and twin pregnancies were more frequent in the epistaxis group (13/104 [12.5%] vs. 125/1924 [6.5%]; p = 0.018).

**Table 1:** Demographics, clinical, and historical characteristics

	Control (N = 1924)	Case- Epistaxis in 3rd Trimester (N = 104)	Total (N = 2028)	P-value
Age	28.00 (23.00, 32.00)	28.00 (23.00, 32.00)	28.00 (23.00, 32.00)	0.742
Ethnicity:				0.967
Jewish	1032 (53.6%)	56 (53.8%)	1088 (53.6%)	
Bedouin	892 (46.4%)	48 (46.2%)	940 (46.4%)	
Parity	1.00 (0.00, 2.00)	1.00 (0.00, 2.00)	1.00 (0.00, 2.00)	**0.016**
Diabetes GDM1	60 (3.2%)	5 (4.8%)	65 (3.2%)	0.356
Diabetes GDM2	25 (1.3%)	1 (1.0%)	26 (1.3%)	0.755
Rhinitis	333 (17.3%)	17 (16.3%)	350 (17.2%)	0.8
Hypertension	62 (3.0%)	3 (2.8%)	65 (3.2%)	0.84
Twin pregnancy	123 (6.5%)	13 (12.5%)	136 (6.8%)	**0.018**
Pre-eclampsia	72 (3.8%)	3 (2.9%)	75 (3.7%)	0.635

Pregnancy outcomes and neonatal data are shown in Table 2. Women with third-trimester epistaxis experienced higher rates of several adverse obstetric outcomes. Blood transfusion was more frequent in the epistaxis group (5/104 [4.8%] vs. 31/1924 [1.6%]; p = 0.014). Preterm labor occurred more often (16/104 [15.4%] vs. 168/1924 [8.7%]; p = 0.022), as did third-trimester vaginal bleeding (8/104 [7.7%] vs. 21/1924 [1.1%]; p < 0.001). Hospitalization duration was slightly longer for women with epistaxis (median 4.5 days [IQR 4.0- 6.0] vs. 4.0 days [3.0- 5.0]; p = 0.002).

**Table 2:** Pregnancy information and neonatal outcomes

	Control (N = 1924)	Case- Epistaxis in 3rd Trimester (N = 104)	Total (N = 2028)	P-value
Blood transfusion	30 (1.6%)	5 (4.8%)	35 (1.7%)	**0.014**
Rhinitis	333 (17.3%)	17 (16.3%)	350 (17.3%)	0.800
Normal delivery	1449 (76.3%)	75 (72.1%)	1524 (76.1%)	0.325
CS	360 (19.0%)	26 (25.0%)	386 (19.3%)	0.129
Eclampsia	3 (0.2%)	0 (0.0%)	3 (0.1%)	0.685
Oligohydramnios	57 (3.0%)	3 (2.9%)	60 (3.0%)	0.945
Polyhydramnios	25 (1.3%)	1 (1.0%)	26 (1.3%)	0.755
PPH	9 (0.5%)	0 (0.0%)	9 (0.4%)	0.482
Preterm labor	166 (8.7%)	16 (15.4%)	182 (9.1%)	**0.022**
Bleeding in 3rd Trimester	22 (1.1%)	8 (7.7%)	30 (1.5%)	** < 0.001**
PROM	219 (11.5%)	12 (11.5%)	231 (11.5%)	1.000
Vacuum delivery	68 (3.6%)	3 (2.9%)	71 (3.5%)	0.720
Hospital days	4.00 (3.00, 5.00)	4.50 (4.00, 6.00)	4.00 (3.00, 5.00)	**0.002**
Delivery week	39.00 (38.00, 40.00)	39.00 (37.00, 40.00)	39.00 (38.00, 40.00)	**0.089**
Preterm group:				** < 0.001**
Very early	25 (1.3%)	1 (1.0%)	26 (1.3%)	
Early	25 (1.3%)	0 (0.0%)	25 (1.3%)	
Late pre-term	292 (15.5%)	32 (31.1%)	324 (16.3%)	
Term	1544 (81.9%)	70 (68.0%)	1614 (81.1%)	
Hemoglobin	10.60 (9.70, 11.60)	10.40 (9.30, 11.45)	10.60 (9.70, 11.60)	0.356
Thrombocytes	221.00 (179.00, 266.00)	205.00 (161.50, 252.00)	220.00 (178.00, 265.00)	**0.055**

Cesarean section rates did not differ significantly (p = 0.129). However, late preterm deliveries were more frequent in the epistaxis group (31.1% vs. 15.5%), with a corresponding reduction in term deliveries (68.0% vs. 81.9%; p < 0.001). Neonatal hemoglobin and platelet counts at delivery were similar between groups.

Clinical details of epistaxis episodes are shown in Table 3. Among women with epistaxis, 21.1% reported a history of prior nosebleeds. Most episodes resolved spontaneously (81.7%), while a few required interventions: nasal packing (6.7%), bipolar cautery (5.6%), or local pressure (5.6%). No woman required hospitalization or surgical intervention for epistaxis.

**Table 3:** Characteristics and management of women with epistaxis during pregnancy (study group)

		Epistaxis patients (N = 104)
History of epistaxis	n (%)	22 (21.1%)
History of epistaxis intervention	n (%)	6 (5.7%)
History of epistaxis admission	n (%)	6 (5.7%)
History of facial trauma	n (%)	4 (3.8%)
Body temperature (C°)	Mean ± SD	36.7
Hemoglobin levels (g/dL)	Mean ± SD	11.5 (± 1.26)
	Q2 [Q1-Q3]	11.5
Thrombocyte levels (10^3^/µL)	Mean ± SD	207.8 (± 62.9)
	Q2 [Q1-Q3]	207
White blood count (10^3^/µL)	Mean ± SD	9.3 (± 2.5)
	Q2 [Q1-Q3]	9.45
INR levels	Mean ± SD	0.96 (± 0.08)
	Q2 [Q1-Q3]	0.95
Management		
Spontaneous control	n (%)	85 (81.7%)
Local pressure	n (%)	6 (5.7%)
Nasal packing	n (%)	7 (6.7%)
Bipolar diathermia	n (%)	6 (5.7%)

**Fig. 1:**
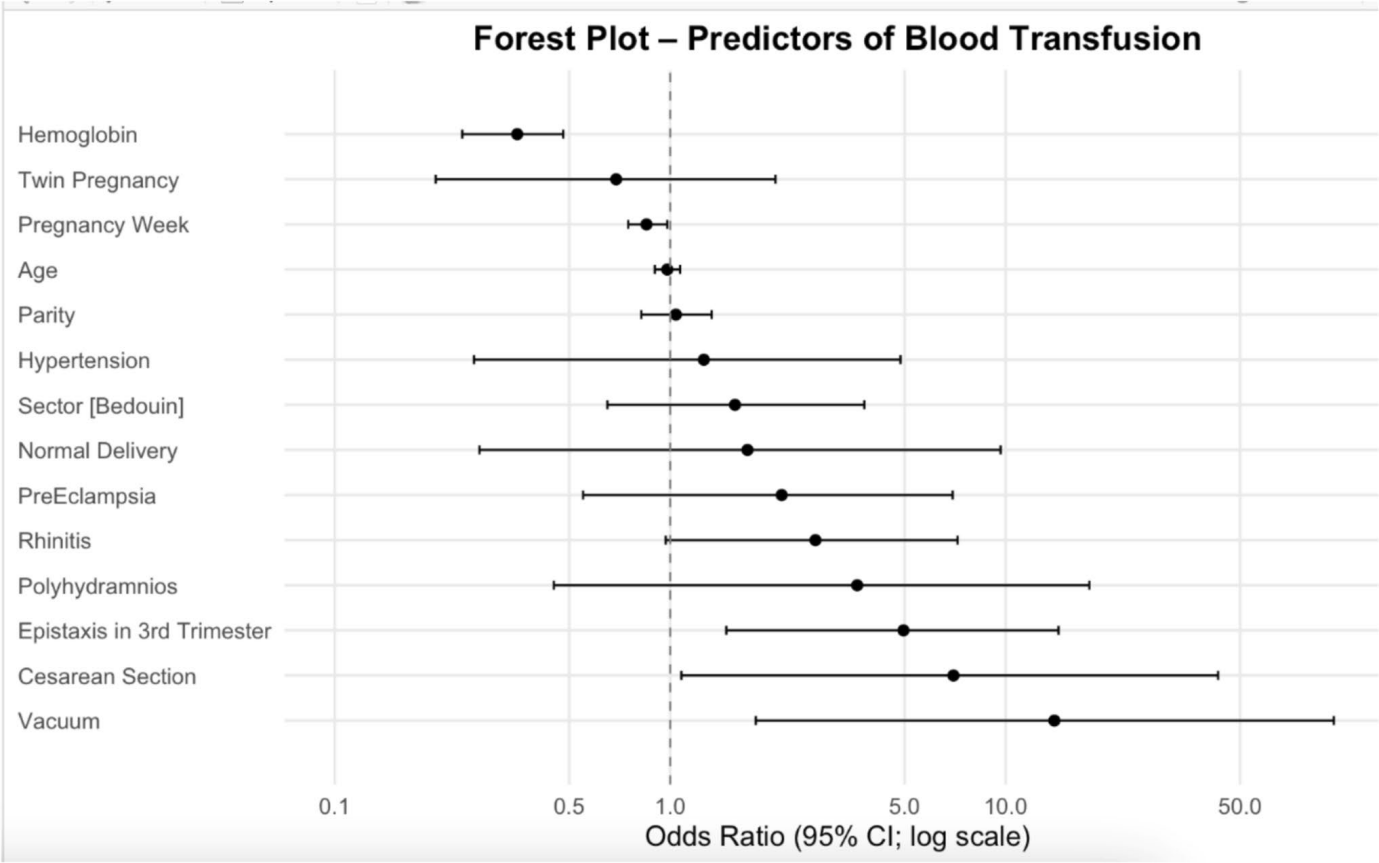
Forest plot of multivariable logistic regression showing predictors of maternal blood transfusion. Epistaxis during the third trimester was an independent risk factor (OR 4.96; 95% CI: 1.47–14.38)

In a multivariable logistic regression examining predictors of blood transfusion (Fig. 1), third-trimester epistaxis was independently linked to higher odds of transfusion (OR 4.96, 95% CI 1.47–14.38; p = 0.005), after controlling for hemoglobin level, delivery mode, and other obstetric factors. Other predictors included lower maternal hemoglobin (OR 0.35 per 1 g/dL decrease; 95% CI 0.24–0.48; p < 0.001), vacuum-assisted delivery (OR 13.97; 95% CI 1.80–95.16; p = 0.008), cesarean delivery (OR 6.99; 95% CI 1.08–42.99; p = 0.037), and earlier gestational age at delivery (OR 0.85 per week; 95% CI 0.75–0.98; p = 0.017). In a different model assessing preterm labor, epistaxis was not an independent predictor (p = 0.254).

## Discussion

Pregnancy and adverse maternal outcomes. Although epistaxis is typically regarded as a self-limiting symptom, our findings suggested it may serve as an early clinical indicator of serious obstetric complications, including the need for blood transfusions, third-trimester vaginal bleeding, and preterm labor. Physiological changes, such as pregnancy-associated rhinitis, increase the risk of epistaxis and exacerbate preexisting sinus conditions. Estrogen further contributes to these issues by promoting glandular activity and vascular engorgement. [7, 8].

In our cohort, most epistaxis episodes were mild, self-limiting, and managed conservatively. Notably, over one-fifth of affected women reported recurrent epistaxis, suggesting an underlying predisposition amplified by pregnancy. While pregnancy induces a hypercoagulable state, compensatory anticoagulant mechanisms typically maintain hemostatic balance [9]. In susceptible women, this balance may be disrupted, potentially increasing the risk of peripartum hemorrhage. These findings underscore the necessity of hematologic assessment and clinical preparedness for this population.

The increased incidence of third-trimester vaginal bleeding in women with epistaxis is noteworthy. While epistaxis is typically attributed to localized vascular fragility, our findings suggest a broader systemic vascular susceptibility [10]. The hyperestrogenic state of pregnancy increases mucosal vascular permeability and fragility, potentially affecting both nasal and genital mucosa. Furthermore, documented cases of pregnancy-related epistaxis due to granulomas or nasal hemangiomas illustrate the profound vascular alterations during gestation [11–13]. Shared endothelial dysfunction or an amplified inflammatory response may contribute to bleeding across multiple mucosal sites [14]. Although the specific etiologies of vaginal bleeding were not analysed, this association warrants further investigation. Consequently, early recognition and closer surveillance of pregnant women with epistaxis may facilitate the identification of those at higher risk for subsequent obstetric complications.

The most clinically significant finding was the independent association between third-trimester epistaxis and blood transfusion requirements, which persisted after adjustment for hemoglobin levels, delivery mode, and other relevant obstetric variables [15, 16]. This observation supports the hypothesis that epistaxis may reflect underlying systemic vascular fragility or a subtle imbalance in hemostasis. While previous studies have suggested an association between epistaxis and postpartum hemorrhage (PPH), our data indicate that epistaxis may predict clinically meaningful hemorrhage even when PPH is not formally documented. Although the association with coded PPH did not reach statistical significance, transfusion requirements were nearly fivefold higher in the epistaxis group. This suggests that the need for blood transfusion may represent a more sensitive marker of significant bleeding than PPH coding alone, reinforcing the importance of heightened clinical vigilance. Nevertheless, given the relatively small number of transfusion events, this finding should be interpreted with caution and regarded as exploratory and hypothesis-generating.

Although univariate analysis associated epistaxis with higher rates of preterm labor, this relationship did not persist in multivariable modelling. This suggests that epistaxis may not independently predispose to preterm labor; rather, it may co-occur with other high-risk factors such as multiple gestation or third-trimester bleeding. Systemic inflammation and vascular fragility, both implicated in epistaxis, may also contribute to premature uterine contractions. Furthermore, bleeding events- whether nasal or vaginal- can stimulate thrombin production, which promotes uterine activity and preterm labor. [17, 18].

Clinically, women presenting with epistaxis may benefit from enhanced surveillance in high-risk obstetric settings. Given the substantial increase in transfusion requirements, clinicians should anticipate potential hemorrhagic events during delivery. Coordinated care among otolaryngologists, emergency physicians, and obstetric teams is essential. Establishing standardized management pathways could further improve maternal and neonatal safety.

This study has several limitations inherent to its retrospective, single-center design. First, underreporting is likely, as mild, self-limiting epistaxis often does not prompt medical consultation. Second, variability in medical documentation and evolving coding practices may have affected the accuracy of certain outcomes, including PPH. Additionally, data on potential confounders- such as smoking status, placenta previa, and inflammatory biomarkers- were inconsistently available. Furthermore, blood transfusion was recorded as a binary variable, limiting more nuanced clinical interpretation. Collectively, these factors may lead to an overrepresentation of severe cases and may limit the generalizability of our findings. Given the limited number of blood transfusion events and the number of covariates included in the multivariable model, the results should be interpreted as exploratory and hypothesis-generating rather than confirmatory.

An additional limitation relates to the timing of hemoglobin measurements. In the epistaxis group, hemoglobin levels were obtained at the time of epistaxis presentation, whereas in the control group, hemoglobin levels were measured at delivery. This difference may limit direct comparability and could have influenced the observed associations.

Future research should elucidate the pathophysiological mechanisms linking epistaxis to adverse obstetric outcomes. Large-scale, multicenter prospective studies are necessary to validate these associations and control for additional confounders. Specifically, exploring the roles of systemic inflammation, endothelial integrity, and coagulation abnormalities may provide valuable insights into this underrecognized clinical entity and facilitate the development of targeted preventive strategies.

## Conclusion

Epistaxis during pregnancy, particularly in the third trimester, is associated with an increased risk of adverse obstetric outcomes, including preterm labor and a higher requirement for blood transfusions. While typically considered benign, epistaxis may serve as a clinical indicator warranting enhanced monitoring and interdisciplinary coordination. Recognizing this association facilitates the early identification and management of potential complications during labor and delivery.

## Supplementary Information

Below is the link to the electronic supplementary material.

Supplementary file1 (DOCX 15 KB)

## Data Availability

No datasets were generated or analysed during the current study.
