# Supplementary material for: Epistaxis during the third trimester of pregnancy is associated with blood transfusion a retrospective case–control study
Source: Arch Gynecol Obstet. 2026 Feb 11;313(1):88. doi: 10.1007/s00404-026-08334-1 (PMC12894171; doi:10.1007/s00404-026-08334-1)
Supplement: Supplementary file 1 — Supplementary file1 (DOCX 15 KB) [file 404_2026_8334_MOESM1_ESM.docx]

Epistaxis During the Third Trimester of Pregnancy is Associated with Blood Transfusion

A retrospective Case-Control Study

Aviad Sapir, MD**^1,2^**, Lior Friedrich, MD**^3^**, Yonathan Osovizky, B.Sc**^2,4^**, Yotam Heilig**^1,2^**, Oded Cohen, MD**^2,5^**, Shay Schneider, MD**^1,2^**

^1^Department of Otolaryngology-Head and Neck Surgery, Soroka University Medical Center, Beer-Sheva, Israel

**^2^**Faculty of Health Sciences, Ben-Gurion University, Beer-Sheva, Israel

^3^Helen Schneider Hospital for Women, Rabin Medical Center- Beilinson Hospital, Sackler Faculty of Medicine, Tel Aviv University, Tel Aviv, Israel

^4^Clinical Research Center, Soroka University Medical Center, Beer-Sheva, Israel

^5^Department of Otolaryngology-Head and Neck Surgery, Samson Assuta Ashdod University Hospital, Ashdod, Israel

**Corresponding author** Aviad Sapir, MD

Department of Otolaryngology-Head and Neck Surgery

Soroka University Medical Center

ISRAEL, Beer Sheva, 84101

Phone number +972 -53-4299591

[aviadsap@gmail.com](mailto:aviadsap@gmail.com)
